# New Insights Into the Role of Seed Oil Body Proteins in Metabolism and Plant Development

**DOI:** 10.3389/fpls.2019.01568

**Published:** 2019-12-10

**Authors:** Qun Shao, Xiaofan Liu, Tong Su, Changle Ma, Pingping Wang

**Affiliations:** Shandong Provincial Key Laboratory of Plant Stress, College of Life Sciences, Shandong Normal University, Jinan, China

**Keywords:** oil body intrinsic proteins, lipid metabolism, hormone signaling, stress responses, plant development

## Abstract

Oil bodies (OBs) are ubiquitous dynamic organelles found in plant seeds. They have attracted increasing attention recently because of their important roles in plant physiology. First, the neutral lipids stored within these organelles serve as an initial, essential source of energy and carbon for seed germination and post-germinative growth of the seedlings. Secondly, they are involved in many other cellular processes such as stress responses, lipid metabolism, organ development, and hormone signaling. The biological functions of seed OBs are dependent on structural proteins, principally oleosins, caleosins, and steroleosins, which are embedded in the OB phospholipid monolayer. Oleosin and caleosin proteins are specific to plants and mainly act as OB structural proteins and are important for the biogenesis, stability, and dynamics of the organelle; whereas steroleosin proteins are also present in mammals and play an important role in steroid hormone metabolism and signaling. Significant progress using new genetic, biochemical, and imaging technologies has uncovered the roles of these proteins. Here, we review recent work on the structural or metabolic roles of these proteins in OB biogenesis, stabilization and degradation, lipid homeostasis and mobilization, hormone signal transduction, stress defenses, and various aspects of plant growth and development.

## Introduction

Lipids are essential for all kingdoms of life because they are involved in a remarkably wide variety of cellular functions, including energy homeostasis, membrane remodeling, and cell signaling (Pyc et al., 2017). The cellular mechanisms of assembling, storing, and supplying lipids by forming intracellular lipid particles are relatively conserved in all eukaryotes, including yeast (Leber et al., 1994), insects (Zhang et al., 2010), mammals (Murphy, 2001), and plants (Bao et al., 2018), as well as prokaryotes (Wältermann et al., 2005);. Traditionally, these lipid particles have had a variety of names, including oil bodies (OB), oleosomes, spherosomes, lipid bodies, lipid droplets (LD), or lipid vesicles. In recent years, the terms OB or LD have been adopted by most laboratories. OBs are present in seeds, leaves, pollens, fruits, flowers, and roots of higher plants (angiosperms), the vegetative and reproductive organs of lower plants, the glands and adipose tissues of mammals, as well as in algae, fungi, nematodes, and bacteria (Huang, 2013; Shimada et al., 2018).

These organelles consist of a densely packed hydrophobic core of neutral lipids surrounded by a phospholipid monolayer decorated by three main classes of OB-associated proteins: oleosins, caleosins, and steroleosins. The acyl moieties of the phospholipid molecules face inward with the hydrophobic triacylglycerol (TAG) in the matrix and the hydrophilic phospholipid head groups in the cytosol (Tzen et al., 1997). OBs in the seeds of plants are generally circular to ovoid with the average diameter of 0.5–2.5 µm, but the diameter of some OBs can be 2–3 times larger than 2.5 µm, such as in legume seeds (Tzen et al., 1993; Song et al., 2017). Recently, 3D reconstruction analysis showed that the volume of most OBs in *Brassica napus* seeds were less than 100 µm^3^ (Yin et al., 2018).

OBs are highly dynamic organelles and are actively involved in many diverse physiological processes including membrane biogenesis supporting organelle or cell growth, diurnal regulation process, hormone signaling, and plant growth and development. OBs also act as a “sink” for toxic fatty acids which can be lethal to the cells (Pyc et al., 2017). The decomposition of OBs by proteases and lipases and the subsequent β-oxidation of the released fatty acids can provide carbon and energy during seed germination and post-germinative growth of the seedlings (Huang, 1996). As a result of their importance in plant physiology, seed OBs have been intensively studied in the past few decades, surpassing our knowledge in nonplant organisms.

The functions of OBs are dependent on three classes of OB intrinsic proteins: oleosins, caleosins, and steroleosins. These distinct structural OB proteins are associated with specific biological functions. The structural or metabolic roles of these proteins in the control of lipid store mobilization, OB degradation, hormone signal transduction, and stress defenses are discussed in this review.

## OB Intrinsic Proteins

### Oleosin, Caleosin, and Steroleosin

OBs in the intact cells of a mature seed never coalesce or aggregate, even after long-term storage, because the entire surface of an OB is covered by proteins (Leprince et al., 1998). The most abundant protein constituents are the structural alkaline proteins termed oleosins. Oleosins are also located in other tissues such as the tapetum and the external surface of pollen grains (Kim et al., 2002). They have a molecular mass of 15–50 kDa, depending on the isoform and plant species in which they occur (Tzen et al., 1990; Jolivet et al., 2009).

The oleosin gene was first cloned from maize (Vance and Huang, 1987). In *Arabidopsis*, 16 oleosin proteins have been identified, including five seed-type oleosins, eight anther-type oleosins, and three seed-and-anther type oleosins (**Table 1**). The most abundant oleosin in *Arabidopsis* seeds is oleosin-1 (OLE1), followed by oleosin-2 (OLE1) (D’Andrea, 2016; Shimada et al., 2008). Seed-type oleosins are involved in regulating both OB size and seed germination. Anther-type oleosins have glycine-rich domains not found in seed-type oleosins and function in stabilizing pollen OBs and forming pollen and the pollen coat (Mayfield et al., 2001; Kim et al., 2002).

**Table 1:** Three OB-associated proteins in *Arabidopsis thaliana*

TAIR locus	Description	Putative function	Reference
**Oleosin**			
AT4G25140	OLE1, OLEO1, OLEOSIN 1	Major seed OB protein, involved in seed lipid accumulation and freezing tolerance of seeds.	van Rooijen et al., 1992; Siloto et al, 2006
AT5G40420	OLE2, OLEO2, OLEOSIN 2	Major seed OB protein, involved in seed lipid accumulation and freezing tolerance of seeds.	Zou et al.,1995; Shimada et al,2008
AT3G27660	OLEO3, OLEOSIN3	Seed OB protein, involved in seed lipid accumulation and OB degradation.	Kirik et al., 1996; Deruyffelaere et al., 2015
AT3G01570	Oleosin 4, OLE4	Major seed OB protein, involved in seed lipid accumulation and freezing tolerance of seeds.	Kim et al., 2002; Shimada et al, 2008
AT5G51210	Oleosin 5, OLE5	Minor seed OB oleosin, a possible role for these oleosins in the control of OB dynamics.	Kirik et al., 1996; Deruyffelaere et al., 2015
AT5G07510	ATGRP14, glycine rich protein 14	A pollen coat protein. No report of function.	Mayfield and Preuss, 2000
AT5G07540	ATGRP17, glycine rich protein 17	A pollen coat protein. No report of function.	de Oliveira et al., 1993
AT5G07530	ATGRP17, glycine rich protein 17	A glycine rich protein containing oleosin domain, found on mature pollen coat, have a role in initiating pollination.	Mayfield and Preuss, 2000
AT5G07520	ATGRP18, glycine rich protein 18	A pollen coat protein. No report of function.	de Oliveira et al., 1993
AT5G07550	ATGRP19, glycine rich protein 19	A glycine rich pollen coat protein. No report of function.	de Oliveira et al., 1993
AT5G07560	ATGRP20, glycine rich protein 20	A glycine rich protein expressed specifically in the florets. No report of function.	Mayfield et al., 2001
AT3G18570	Oleosin family protein	A protein expressed in both maturing seeds and florets. No report of function.	Kim et al., 2002
AT2G25890	Oleosin family protein	A protein expressed in both maturing seeds and florets . No report of function.	Kim et al., 2002
AT1G48990	Oleosin family protein	A protein expressed in both maturing seeds and florets. No report of function.	Kim et al., 2002
AT5G07600	Oleosin family protein	A oleosin expressed specifically in the florets (tapetum). No report of function.	Kim et al., 2002
AT5G61610	Oleosin family protein	A oleosin expressed specifically in the florets (tapetum). No report of function.	Kim et al., 2002
**Caleosin**			
AT4G26740	AtCLO1, ATPXG1, ATS1, CLO1	A caleosin in seed OBs. Catalyze hydroperoxide-dependent mono-oxygenation reactions and sensitive to some hormones.	Naested et al., 2000; Hanano et al., 2006
AT5G55240	AtCLO2, ATS2, ATPXG2	A seed caleosin with peroxygenase activity has roles in dormancy or germination of seeds.	Hanano et al., 2006
AT2G33380	AtCLO3, ATPXG3, RD20	A caleosin expressed in various organs acts as a peroxygenase involved in oxylipin metabolism during stress and sensitive to various stresses.	Aubert et al., 2010; Blée et al., 2014
AT1G70670	AtCLO4, ATPXG4	A stress-responsive and caleosin-like protein mainly expressed in leaf and was sensitive to some stresses in root and cell culture.	Hanano et al., 2006; Kim et al., 2011; Blée et al., 2012
AT1G23240	AtCLO5, ATPXG5	A caleosin was mainly expressed in bud.	Hanano et al., 2006
AT1G70680	AtCLO6	Caleosin family protein. No report of function.	Shen et al., 2014
AT1G23250	AtCLO7	Be without conserved EF-hand and might lost the ability to bind calcium	Shen et al., 2014
AT5G29560	AtCLO8	No report	Shen et al., 2014
**Steroleosin**			
AT5G50600 / AT5G50700	AtHSD1	A hydroxysteroid dehydrogenase in seed OBs acts as a NADP^+^-dependent 11β-,17β-hydroxysteroid dehydrogenase/17β-ketosteroid reductase.	Jolivet et al., 2004; d’Andréa et al., 2007
AT3G47350	AtHSD2	A putative hydroxysteroid dehydrogenase (HSD)	Li et al., 2007
AT3G47360	AtHSD3	A putative hydroxysteroid dehydrogenase (HSD).	Li et al., 2007
AT5G50590/AT5G50690	AtHSD4	A putative hydroxysteroid dehydrogenase (HSD).	Li et al., 2007; Baud et al., 2009
AT4G10020	AtHSD5	A putative hydroxysteroid dehydrogenase (HSD).	Li et al., 2007
AT5G50770	AtHSD6	A putative hydroxysteroid dehydrogenase (HSD).	Li et al., 2007

Another major group of OB structural proteins are the caleosins. Caleosin was first reported (named as Sopl) as a minor constituent in purified OBs from sesame (*Sesamum indicum*) seeds. Their name arose from their ability to bind calcium and their oleosin-like structures (Chen et al., 1998; 1999). Eight caleosin genes (*AtCLO1*-*AtCLO8*) have been found in the *Arabidopsis* genome (**Table 1**) (Hanano et al., 2006; Shen et al., 2014). Among them, *AtCLO1* and *AtCLO2* are preferentially expressed in developing embryos and seeds during seed maturation and the first days following germination. *AtCLO5* expression level is low but detectable in buds. *AtCLO3* is mainly expressed in above-ground tissues, whereas *ATCLO4* is expressed in the vascular bundles in all major plant tissues, as well as in guard cells and germinating seeds (Aubert et al., 2010; Kim et al., 2011; Shen et al., 2014). Expression data for the other caleosin genes are still scarce.

In contrast with oleosins, caleosins are present in more primitive species, such as fungi and single-celled algae (Charuchinda et al., 2015), whereas oleosins are only present in more recent higher plant species (Huang et al., 2009). Because of these observations, oleosin may have been derived from caleosin, which may represent a more ancient structural OB protein in plants, and has become more specifically associated with OB formation and maintenance (Jiang and Tzen, 2010).

The earliest identified plant steroleosins were Sop2 and Sop3 from sesame, named steroleosin-A and -B, respectively. They are homologous proteins with sterol-binding and sterol-coupling dehydrogenase activity (Chen et al., 1998; Lin et al., 2002). Later, the homologous steroleosin encoded by At5g50600 was purified from *Arabidopsis* seed OBs (Jolivet et al., 2004). This protein, with a high degree of similarity to Sop2, exhibits hydroxysteroid dehydrogenase (HSD) activity and belongs to the short-chain steroid dehydrogenase reductase superfamily (SDR) which is involved in sterol-regulated signal transduction in diverse organisms. As a result, this protein is named AtHSD1 (d’Andréa et al., 2007). There are eight putative steroleosin homologs in the *Arabidopsis* genome, including two identical copies of *AtHSD1*, two identical copies of *AtHSD4*, and four other homologs. Sequence alignment showed that the promoter, coding sequence, and terminator of two copies of the AtHSD1 gene, and two copies of the AtHSD4 gene are completely identical at the nucleotide level (**Table 1**) (Li et al., 2007).

Unlike oleosin and caleosin proteins which are specific to plants, OB-associated sterol dehydrogenases are also present in mammals. However, phylogenic analysis indicates that plant 17β-HSDs display only 24% identity with those corresponding sequences in mammals. These enzymes have been demonstrated to be important in steroid hormone metabolism and signaling in both plants and mammals (d’Andréa et al., 2007).

### Structure of OB-Associated Proteins

**Figure 1:**
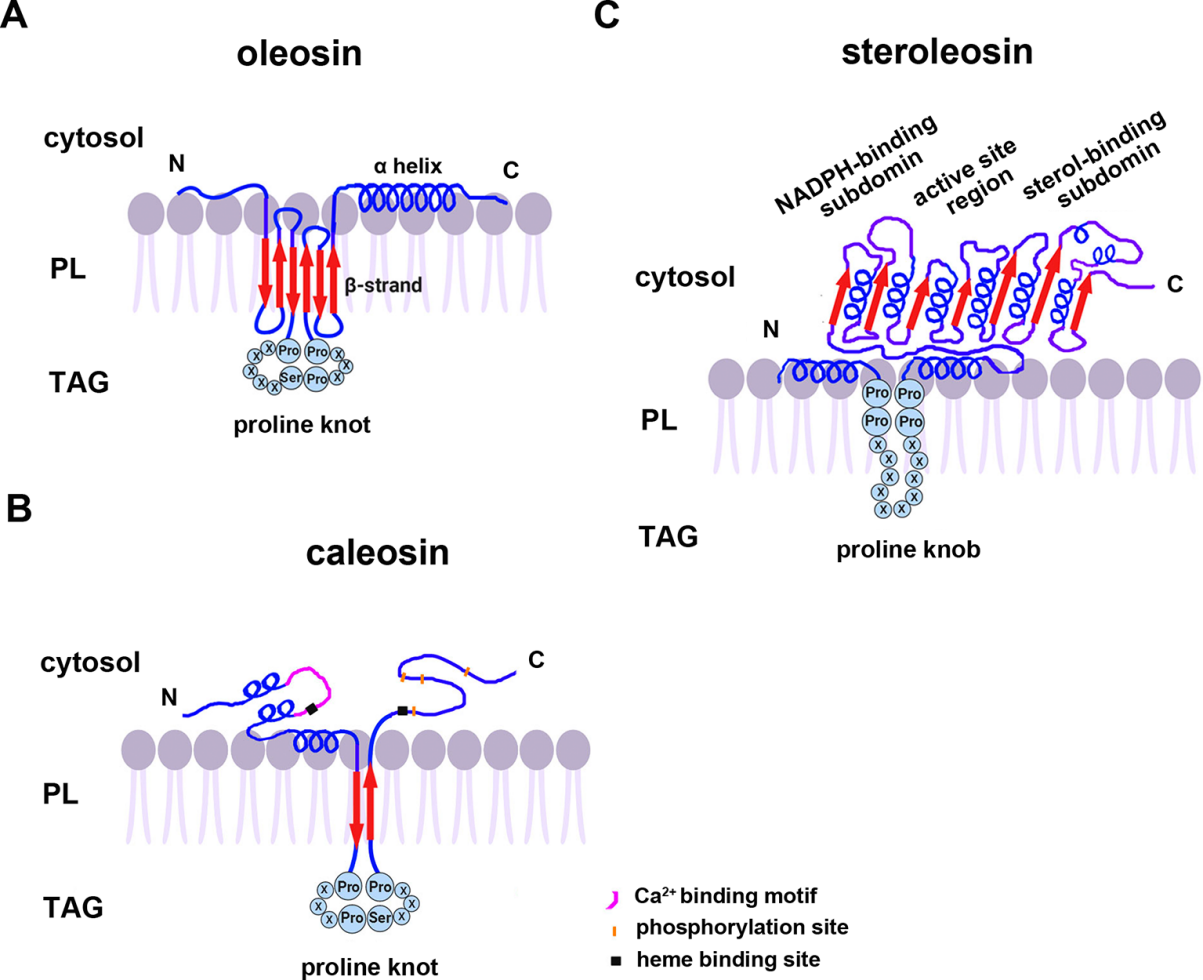
A model of the secondary structure organizations of **(A)** oleosin, **(B)** caleosin, and **(C)** steroleosin on the surface of plant seed oil bodies. Predicted structures of the proline knot or proline knob motif are shown in all three proteins. The model is mainly based on information from Chen et al. (1999); Hanano et al. (2006); d’Andréa et al. (2007), and Tzen (2012). TAG, Triacylglycerol; PL, phospholipid.

All oleosins and caleosins have three regions: a hydrophilic N-terminal domain, a specific and highly conserved hydrophobic central domain and a hydrophilic C-terminal α-helical domain (Tzen and Huang, 1992). The central domain forms a hairpin-like structure composed of antiparallel β strands connected by a proline knot motif and is responsible for OB localization. The N- and C-terminals are exposed to the cytoplasm, and the central domain anchors the protein in the OB membrane (Huang, 1996; Huang and Huang, 2017). In contrast to oleosin, caleosin has a significantly larger N-terminal hydrophilic domain containing an EF-hand calcium binding motif and several potential phosphorylation sites within the C-terminal hydrophilic domain. Both the N- and C-terminal regions of caleosin also contain heme-binding sites with conserved histidine residues that together coordinate the binding of heme prosthetic groups (**Figure 1**) (Hanano et al., 2006; Chapman et al., 2012).

Unlike oleosin and caleosin, steroleosin possesses a distinctive structure containing an N-terminal hydrophobic OB-anchoring segment and a soluble sterol-binding dehydrogenase/reductase domain, located in the cytosol. A unique proline knob motif is in the middle of the steroleosin N-terminus, corresponding to the hydrophobic segment that is presumably responsible for association with the OB phospholipid monolayer. The core structure of the sterol-binding dehydrogenase/reductase domain contains a conserved NADPH-binding subdomain, a NSYK conserved active site region and a divergent sterol-binding subdomain (**Figure 1**) (Lin et al., 2002; d’Andréa et al., 2007).

Cotranslational and posttranslational modifications of OB-associated proteins may elevate their structural stability and prevent ubiquitination and degradation of OBs. Mass spectrum analyses showed that the first methionine in the N-termini of nascent oleosin and caleosin isoforms from sesame seed OBs is removed and the following alanine is acetylated. N-terminal-acetylation is catalyzed by several distinct N-terminal acetyltransferases (NATs) in eukaryotes—NatA-NatF. N-termini with small amino acid residues in the second position (such as Met Ser-, Met Ala-, like oleosin and caleosin, Met Thr-, Met Val-), are mostly processed by methionine aminopeptidase (MAP), and the resulting newly generated N-termini may be acetylated by NatA (Arnesen, 2011; Ree et al., 2018 ). However, candidates for the proposed acetyltransferase activities that modify the N-termini of oleosins have yet not been reported. Additionally, deamidation of a glutamine residue is also found in the N-terminus of oleosin. Deamidation of glutamine residues have been considered to be the most common post-translational modification occurring in living systems and to be nonenzymatic reactions (Li et al., 2010). This posttranslational modification introduces more negative charges to the protein surface and may reinforce OB stability by preventing aggregation under physiological conditions. Amino acid sequence analysis shows that both steroleosin isoforms (Sop2 and Sop3) possess a free initial methionine residue at their N-termini. They also possess an N-terminal sequence responsible for endoplasmic reticulim (ER) targeting *via* the signal-recognition particle (SRP) dependent pathway which anchors to OBs. No ER targeting signal sequences are present in the N-termini of caleosin or oleosin isoforms. Because of this signal sequence, no posttranslational cleavage or modification occurs in the N-termini of the mature steroleosins (Lin et al., 2005).

## Oleosins Function During Seed Maturation and Germination

Oleosins are only found in plants, including green algae (Huang, 2013). However, in mammals and insects, a different set of abundant OB proteins, such as perilipins (PLINs) (Sztalryd and Kimmel, 2014) that are absent in plants, serve similar functions. Although the lipid bodies in yeast, such as *Saccharomyces cerevisiae*, are functionally similar to OBs in plant seeds, no oleosin homologs are present (Jacquier et al., 2013).

**Figure 2:**
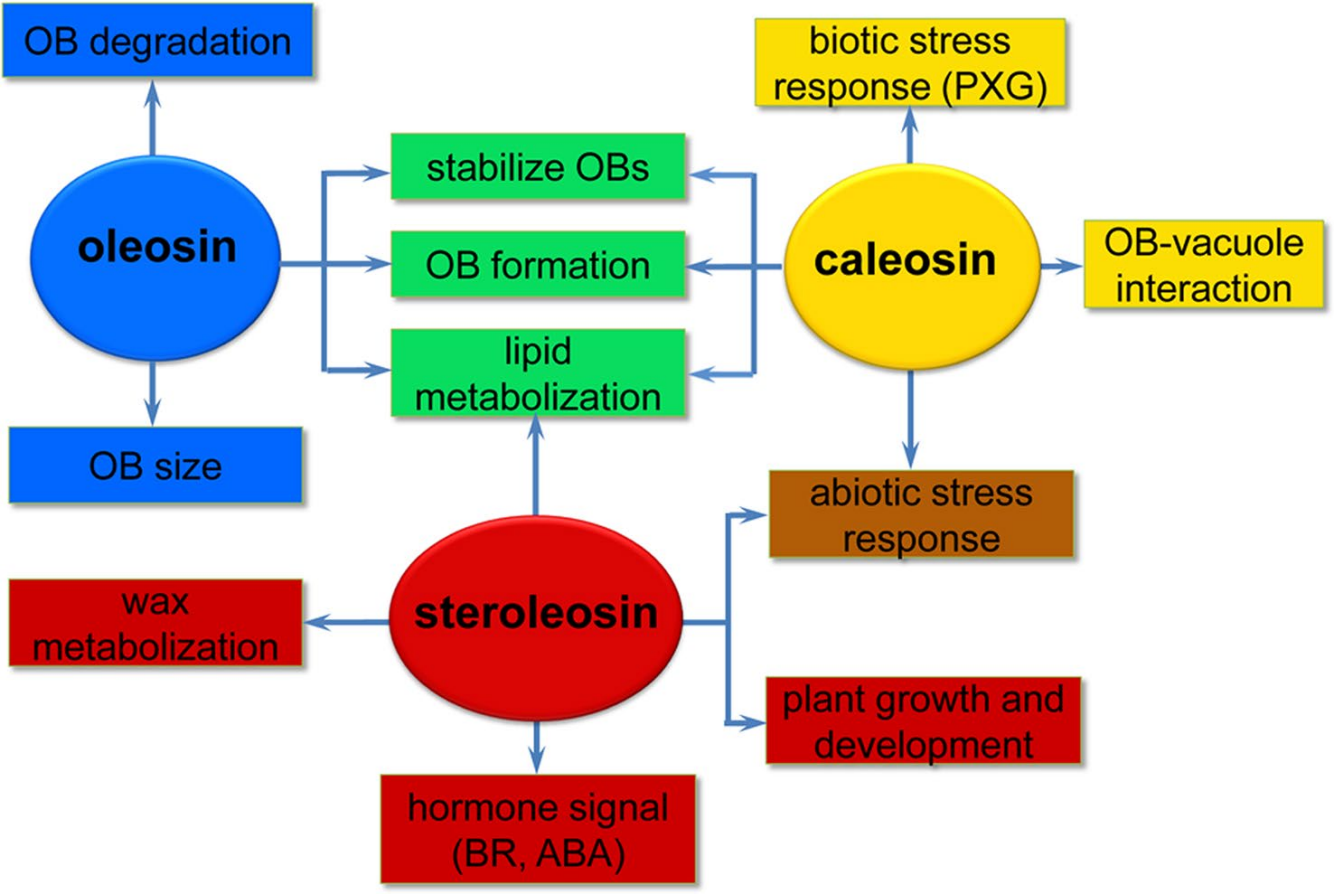
A simplified overview of the putative roles of the three oil body (OB) intrinsic proteins in various processes of plant development. Oleosins (blue) mainly act as structural proteins stabilizing OBs and are involved in lipid metabolization during seed germination and seedling growth. Caleosins (orange) play important roles in biotic and abiotic stress responses through their peroxygenase (PXG) activities. Steroleosins (red) are involved in regulating plant growth and development mainly *via* an unknown mechanism by which the proteins interact with hormones such as brassinosteroids (BRs) and abcisic acid (ABA). Green indicates processes that both oleosin and caleosin are involved in. Brown indicates processes that both caleosin and steroleosin are involved with.

Oleosins have a variety of confirmed functions. They contribute to the stability and resolubility of OBs during seed (and pollen grain) desiccation, regulate OB size and viability in overwintering seeds, and are important for lipid mobilization during seed germination (**Figure 2**) (Schmidt and Herman, 2008; Shimada et al., 2008; Wu et al., 2010; Miquel et al., 2014).

### Oleosins Affect the Stability and Size of OBs

Oleosins stabilize OBs by creating a negative charge on the OB surface which prevents OB coalescence due to steric hindrance and electrical repulsion (Tzen and Huang, 1992). OB reconstitution showed that oleosins are essential to avoid coalescence and important for maintaining the physical stability of OBs (Deleu et al., 2010). Oleosin concentrations have been reported to correlate with OB size in several oil plants including rape, mustard, cotton, flax, maize, peanut, sesame, olive, and avocado (Tzen et al., 1993; Ting et al., 1996). The size of OBs is inversely proportional to the concentration of oleosin: high-oil seeds of maize with relatively low oleosin levels have large OBs, while low-oil seeds of maize with high oleosin levels have small OBs (Ting et al., 1996). Similarly, the avocado mesocarp cell, which does not express oleosin, has only one very large OB (20µm in diameter) (Platt-Aloia and Thompson, 1981). Several studies in *Arabidopsis* and rice have shown that oleosin depletion caused by RNA interference (RNAi) leads to the appearance of unusually large and structurally abnormal OBs (Siloto et al., 2006; Miquel et al., 2014).

In mature *Arabidopsis* seeds, OLE1 and OLE2 accumulate at higher levels than OLE3 and OLE4, whereas OLE5 has the lowest abundance (Siloto et al., 2006; Miquel et al., 2014). Shimada et al. investigated the physiological function of oleosins using an oleosin-deficient mutant series of *A. thaliana* and found that oleosin levels affected germination and the freezing tolerance of seeds. Among the mutants used, the double *Arabidopsis* mutant *ole1 ole2* has the lowest levels of oleosins and hardly germinates under normal conditions. In addition, freezing treatment followed by imbibition at 4°C accelerates the fusion of OBs and generates acentric nuclei (irregularly shaped nuclei found on the periphery of seed cells) leading to seed mortality and inhibition of seed germination in the *ole1 ole2* mutant. Thus, oleosins increase seed viability and maintain seed germination by preventing abnormal fusion of OBs during overwintering (Shimada et al., 2008).

### Oleosins Regulate Lipid Metabolization

Oleosins play an important role in regulating the biosynthesis, metabolization, and mobilization of lipids during seed maturation and germination (Parthibane et al., 2012). Increasing evidence suggests oleosins affect the lipid levels of plant organs. In *S. cerevisiae*, phosphatidic acid phosphohydrolase (PAH1) catalyzes the rate-limiting step for TAG formation, the dephosphorylation of phosphatidic acid to diacylglycerol (DAG), which play a direct role in OB biogenesis (Skinner et al., 2009). OB numbers of yeast cells lacking PAH1 (*pah1* mutant) reduce by more than 60%. PAH1, probably working through DAG, controls the formation of OBs (Adeyo et al., 2011). Ectopic expression of *A. thaliana* oleosin1 (OLE1) in *pah1* cells not only increases the levels of newly synthesized neutral lipids including TAG and steryl esters (STE), but also increases OB formation. OBs are formed from membranes enriched in neutral lipids by promoting the sequestration of neutral lipids within the ER bilayer and protecting the TAG pool from hydrolysis by lipases (Jacquier et al., 2013). It is well known that diacylglycerol *O*-acyltransferase (DGAT1) is a critical enzyme involved in TAG synthesis *via* the acyl-CoA-dependent pathway in developing seeds (Chapman and Ohlrogge, 2012). Winichayakul et al. coexpressed *Arabidopsis* DGAT1 and a synthetically stabilized oleosin (cysteine [Cys]-oleosin) from sesame seeds in *Arabidopsis* and *S. cerevisiae*. Both transgenic plants and yeast cells produced significantly increased neutral lipid levels in vegetative organs and yeast cells than in the respective wild type cells or when expressing DGAT1 alone. The increasing neutral lipids then elevates CO_2_ levels in the chloroplast, leading to a higher CO_2_ fixation rate and an increase in biomass production (Winichayakul et al., 2013). Overexpression of *Arabidopsis* seed OLE1 induces elevated leaf TAG levels in transgenic *Arabidopsis* plants. TAG content is increased up to sevenfold compared to wild type (Fan et al., 2013). Vanhercke et al. also obtained the similar results. They found that simultaneous expression in tobacco of the *A. thaliana* transcription factor WRI1, DGAT, and the *S. indicum OLEOSIN* gene caused TAG levels in tobacco leaves to dramatically increase (more than 15% of the dry weight) without a detrimental effect on plant development or seed viability (Vanhercke et al., 2014). Four *Brassica napus* oleosin genes were overexpressed in *Arabidopsis* increased the linoleic acid content (13.3% at most) and the seed weight. In the meanwhile, the eicosaenoic acid content decreased by 11% in seeds of transgenic lines (Chen et al., 2019). The experimental evidence confirmed that oleosins play important roles in regulating lipid metabolization.

### Oleosins Play an Important Role in OB Formation and Degradation

In seeds, oleosins are also involved in OB formation and degradation (Schmidt and Herman, 2008; Quettier and Eastmond, 2009). During OB biogenesis, oleosin is first integrated into the ER membrane before it is targeted to the OBs (Ting et al., 1997; Jacquier et al., 2013).

OBs are structurally and functionally conserved across species. Several studies demonstrated that plant oleosins from maize (*Zea mays* L.), sunflower, and *B. napus* are correctly targeted to endogenous OBs when heterologously expressed in *S. cerevisiae* or mammalian cells (Ting et al., 1997; Beaudoin et al., 2000; Hope et al., 2002). Thus, because of the convenience of using yeast cells, expression of plant oleosins in yeast cells is helpful to investigate the possible function of these proteins in neutral lipid homeostasis and OB biogenesis. In *S. cerevisiae*, four enzymes, including acyl-CoA: sterol acyltransferases (Are1 and Are2) which produce STE, lecithin cholesterol acyltransferase (LCAT)-related protein (Lro1), and acyl-CoA: diacylglycerol acyltransferase (Dga1) which produces TAG, are involved in synthesis of neutral lipids and LD biogenesis (Oelkers et al., 2002; Czabany et al., 2007). In quadruple yeast mutant cells lacking OBs due to the deletion of all four genes, some OB-localized membrane proteins, such as Erg6 (an enzyme of the ergosterol biosynthetic pathway) or Dga1, are mislocalized in the ER bilayer. Induction of neutral lipid synthesis in these mutants leads to the formation of OBs and a concomitant relocalization of the mislocalized OB proteins from the ER onto OBs (Jacquier et al., 2011). Similarly, GFP-tagged versions of OLE1 from *A. thaliana* were expressed in *S. cerevisiae* and showed proper targeting of the protein to OBs. In wild-type yeast cells, GFP-OLE1 induces OB formation, but in yeast mutant cells lacking OBs GFP-OLE1 localizes to the ER membrane and is significantly less stable and rapidly degraded (Jacquier et al., 2013). These observations suggest that oleosins have high affinity for neutral lipids and phospholipids, probably due to the extraordinary architecture of oleosins which is ideal for localization within OBs.

During plant seed germination and seedling growth, oleosins are hydrolyzed by endogenous proteases as the initial step in lipase-induced TAGs mobilization. The hydrolysis of oleosins has been observed in many plant species. To date, the reported enzymes involved in this process include the ubiquitin/proteasome system, the thioredoxin-regulated cysteine protease, and the aspartic protease, among others.

In plants, degradation of most intracellular proteins is *via* ubiquitination of the respective proteins and subsequent digestion by the proteasome (Sorokin et al, 2009). Ubiquitination of oleosins was first reported in young sesame seedlings. Ubiquitinated oleosin and caleosin were detected after seed imbibition by mass spectrometric analyses and further immunological detection using antibodies against ubiquitin (Hsiao and Tzen, 2011). Using proteomic and immunochemical approaches, Deruyffelaere et al. revealed the physiological regulation of oleosin ubiquitination during seed germination and defined the topologies of the ubiquitin attached to oleosins. OLE1-OLE5 are hydrolyzed sequentially by proteases just prior to lipid degradation in germinated seeds, concomitant with several posttranslational modifications of the oleosins. During this process, OLE5 degraded first, followed by OLE2 and OLE4, and then OLE1 and OLE3. The OLE5 and 8 kDa proteolytic fragment of OLE2 are phosphorylated, while OLE1-OLE4 are ubiqutinated at the onset of lipid degradation. The ubiquitination topology of the oleosins is complex and differs for the various oleosins, suggesting distinct specific degradation pathways (Deruyffelaere et al., 2015). Recently, two research teams presented important insights into the mechanism regulating the extraction and turnover of oleosins in plants. Two key components, PUX10 (a member of the plant ubiquitin regulatory X (UBX)-domain containing protein family) and CDC48A (the AAA ATPase, Cell Division Cycle 48) were found. PUX10 localizes to OBs and binds to the ubiquitinated oleosins *via* its hydrophobic domain and interacts with ubiquitin and CDC48A *via* its UBA and UBX domains, respectively. As an adaptor, PUX10 recruits CDC48A to ubiquitinated oleosins, leading to dislocation of oleosins from OBs *via* the segregase activity of CDC48A (Deruyffelaere et al., 2018; Kretzschmar et al., 2018).

Trx h (thioredoxin h) activates a thiol-protease to degrade the oleosin coat of OBs in sunflower (*Helianthus annuus* L.) seedlings (Babazadeh et al., 2012). In soybean, series analysis of proteases, including analyzing the specificity, optimal pH, and temperature, suggest that OB extrinsic proteins, probably two thiol proteases of the papain family, Bd 30K and P34, originated during seed maturation, are responsible for the hydrolysis of 24 and 18 kDa oleosins (Chen et al., 2014). Similarly, a two-chain (32 and 9 kDa) aspartic protease was identified from the crude extract of peanut OBs. This enzyme shows high affinity for OBs and hydrolyzes both OB intrinsic proteins, such as oleosin, caleosin, steroleosin, and extrinsic proteins (Chen et al., 2018). However, these studies did not provide direct evidence for the interaction between endogenous proteases and oleosins, and the proteases for the oleosin hydrolysis were also not well-known.

Oleosins are involved in several OB functions. In addition to being the main structural protein to maintain OB stability, oleosins are important for the biogenesis and dynamics of the organelle. However, the precise mechanism underlying oleosins’ functions, especially in OB degradation, and how oleosins interact with other proteins require further, more extensive research.

## Caleosin Functions in Plant

As the OB-associated proteins, besides being confined to the OB surface in plant seeds, caleosins have also been detected in vegetative tissues where they are associated with the endoplasmic reticulum, the vacuole and the envelope of chloroplasts (Hernandez-Pinzon et al., 2001; Carter et al., 2004; Partridge and Murphy, 2009). These diverse localizations suggest that distinct caleosins may fulfill various physiological functions in plant growth, development, and regulation of plant-environment interactions (**Figure 2**).

### Caleosins Affect the Stability of OBs and Lipid Metabolization

Caleosins are also considered structural stabilizers of OBs. Analyses of lower plant species such as cycad (*Cycas revoluta*) confirm that OBs from megagametophytes are primarily associated with caleosin, while oleosin is absent (Jiang et al., 2009; Jia ng and Tzen, 2010). In another case, stable artificial OBs composed of TAG and phospholipids can be stabilized by the addition of caleosin alone, in the absence of oleosin (Chen et al, 2004). Moreover, knockdown of a 24 kDa oleosin using RNAi in soybean seed OBs is accompanied by an increased caleosin content, reflecting a compensatory mechanism of caleosin to maintain OB integrity (Schmidt and Herman, 2008).

Caleosin is involved in lipid metabolization and biogenesis of OBs. In *Arabidopsis*, AtCLO1 is exclusively expressed during seed development and its expression is not affected by exogenous abcisic acid (ABA) or osmotic stresses in vegetative tissues, indicating its seed-specific roles (Naested et al., 2000). Studies from two *Arabidopsis Atclo1* mutants provide evidence for a role of caleosin in lipid degradation and trafficking in OBs during seed germination. Embryos from germinating *Atclo1* mutant seeds exhibit a significant delay in the breakdown of storage lipid and display distorted vacuole morphology, abnormal internalization of vacuole membranes, and a significant decrease in OB-vacuole interactions. These results indicate that caleosin participates in the interactions between OBs and vacuoles that affect break-down of OBs during germination (Poxleitner et al., 2006).

Heterologous AtCLO1 expression in yeast causes accumulation of OB neutral lipids, resulting in larger and more abundant OBs containing more fatty acids and steryl esters (Froissard et al., 2009). However, the impact of heterologous expression of caleosin versus oleosin in yeast is distinct, with oleosin expression inducing normal OB formation, and expressed oleosins were properly targeted to OBs (Ting et al., 1997; Jacquier et al., 2013). Thus, caleosin and oleosin play important but non-redundant roles in OB biogenesis.

### The Putative Role of Caleosins in Stress Responses

The Ca^2+^-binding EF-hand motif in the N-terminus of caleosins is indicative of the protein’s role in environmental adaptation. Calcium not only serves as a necessary nutrient for plant growth and development but also acts as one of the most important secondary messengers in many processes, such as cell division, apoptosis, polarity formation, photosynthesis, and stress resistance (Yang et al., 2017; Zheng et al., 2017; Jin et al., 2018). AtCLO3 [also designated RESPONSIVE TO DEHYDRATION20 (RD20)], a leaf caleosin isoform with Ca^2+^-dependent protein kinase activity, is strongly induced by many abiotic stresses such as drought, high salinity, and ABA, suggesting this protein may participate in stress signal transduction (Aubert et al, 2010; Aubert et al., 2011; Blée et al., 2014). Consistent with this idea, *AtCLO3* knock-out plants show enhanced stomatal opening and reduced drought tolerance indicating that AtCLO3 plays an important role in the drought response by controlling stomatal aperture (Aubert et al., 2010). The expression of AtCLO4, another OB-associated caleosin in *Arabidopsis*, is down-regulated by exogenous ABA exposure and salt stress. The *atclo4* mutant is hypersensitive to ABA during seed germination and shows increased drought tolerance in the adult stage. Exogenous ABA treatment in the *atclo4* mutant leads to increased expression of some ABA-dependent regulatory genes, such as *ABF3* and *ABF4*. Experimental data suggest that AtCLO4 functions as a negative regulator in ABA signaling and has important roles in the plant’s response to environmental stresses, in addition to its possible roles in seed development and germination in *Arabidopsis* (Kim et al., 2011).

The presence of several heme-binding motifs in caleosin indicates that caleosins can bind heme and possess peroxygenase (PXG) functionality. In plant, peroxygenases catalyze hydroxylation and epoxidations of unsaturated fatty acids by transferring one oxygen atom from a hydroperoxide to the corresponding substrate, thus oxidizing it (Blée et al., 2012). Peroxygenase is also involved in the biosynthesis of plant oxylipins, a large family of oxidized fatty acids and metabolites originating from polyunsaturated fatty acids (PUFAs). Formation of oxylipins is mainly initiated by α-dioxygenases (α-DOX) or lipoxygenases (LOX) (Lòpez et al., 2011). α-DOX converts PUFAs into highly reactive 2-hydroperoxyoctadecatrienoic acids, which can be converted into the corresponding 2-hydroxyoctadecatrienoic acid or decomposed nonenzymatically into CO_2_ and shortened aldehyde derivatives (Hamberg et al., 2003). LOX catalyzes oxygenation of unsaturated fatty acids, yielding the corresponding fatty acid hydroperoxides, which are then reduced by peroxygenases into the corresponding fatty acid hydroxides (FAOH). Such oxylipins play significant roles in plant defense against pests and pathogens by inducing defense genes, regulating cell death or acting directly through their antimicrobial properties (Prost et al., 2005; Lòpez et al., 2011). Some caleosins, such as AtCLO1-4 from *Arabidopsis*, have been identified as calcium-binding heme-oxygenases with peroxygenase activity (Partridge and Murphy 2009; Kim et al., 2011).

Purified PXG (a caleosin from oat seed OBs) and crude extracts of yeast expressing the recombinant AtCLO1, AtCLO2, and EFA27 (a caleosin from rice) proteins were all able to perform co-oxidation reactions typical of peroxygenase, such as oxidizing thiobenzamide to its sulfoxide and oleic acid into 9,10-epoxy-stearate (Hanano et al., 2006).

In addition to induction by abiotic stresses, the expression of AtCLO3 is also enhanced by pathogens, suggesting a role in biotic stress responses as well (Shimada et al., 2014). The recombinant protein obtained from *S. cerevisiae* expressing the peroxygenase AtCLO3 was confirmed to possess hydroperoxide reductase activity, leading to the formation of endogenous FAOH from hydroperoxides of unsaturated fatty acids. Arabidopsis plants overexpressing AtCLO3 also accumulate 13-hydroxy-9,11,15- octadecatrienoic acid, a linolenate-derived hydroxide. These FAOHs confer tolerance to oxidative stress by decreasing the accumulation of reactive oxygen species (ROS) and minimizing cell death (Blée et al., 2014). Microarray analysis in wild type plants showed that AtCLO3 is coexpressed with genes involved in the biosynthesis of very long chain fatty acids (VLCFA), components of seeds, and cuticular waxes. This experiment revealed that, compared to control plants, *Arabidopsis* overexpressing AtCLO3 exhibit an increased proportion of VLCFA in fatty acid composition of the seeds and contain higher amounts of alkanes and aldehydes in leaf cuticular waxes. As a result, altering the levels of these leaf cuticle wax components increases resistance to the fungus *Alternaria brassicicola* (Hanano et al., 2015). Shimada et al. observed that α-DOX1 (a leaf OB protein with α-dioxygenase activity) and AtCLO3 work together to catalyze coupled reactions to produce 2-hydroxyoctadecanoic acid (2-HOT), which has antifungal activity against members of the genus *Colletotrichum*. Infection with *C.higginsianum* induces 2-HOT production and promotes formation of AtCLO3 and α-DOX1-positive OBs in the area around the site of infection. This study provides evidence of leaf OB function in plant defense *via* production of a phytoalexin under pathological conditions (Shimada et al., 2014; Shimada and Hara-Nishimura, 2015). Thus, the peroxygenase activities of these proteins were involved in the oxylipin signaling pathway and plant defense responses.

To date, among the Arabidopsis caleosin-like genes, possible roles have only been reported for AtCLO1, AtCLO3, and AtCLO4. The roles of the other caleosins remain largely uncharacterized.

## HSD Expression and Function in Plant

Compared with oleosin and caleosin proteins, HSDs are only minor components of OBs in oilseed plants and thus less emphasis has been placed on investigating steroleosins (d’Andréa et al., 2007). Unlike oleosin or caleosin, steroleosin does not play a key role in maintaining the structural stability and integrity of OBs. When stabilized only by recombinant steroleosin fusion proteins (F-steroleosin or DS steroleosin), artificial pine OBs lacking oleosin and caleosin are relatively unstable and larger than native OBs (Pasaribu et al., 2016). However, steroleosins play important roles in plant development and stress responses *via* the enzymatic activity of the HSDs and hormone signaling (**Figure 2**).

### Enzyme Activity and Substrate Analysis of HSDs

OBs from different forms of life have been reported to possess HSD activity and to be involved in the metabolism of steroids. However, there have only been a few reports on the presence of active HSDs in plants. Studies suggest that *Arabidopsis* and *S. indicum* seed OBs contain 11β- and 17β-HSD activities carried out by AtHSD1, and Sop2 and Sop3, respectively (Lin and Tzen, 2004; d’Andréa et al., 2007; Li et al., 2007).

Overexpressed Sop2 and Sop3 proteins are capable of oxidizing estradiol and corticosterone, thus exhibiting 17β- and 11β-HSD activities. However, they possess different sterol selectivities and NADP^+^ specificities. Sop2 exhibits higher dehydrogenase activity to estradiol than to corticosterone in the presence of either NADP^+^ or NAD^+^, and higher activity is detected using NADP^+^ than NAD^+^ as a cofactor when comparing the the same sterol substrate. In contrast, Sop3 is active and shows similar dehydrogenase activities to both examined sterols, but only in the presence of NADP^+^. Therefore, these two steroleosins may conduct different biological functions during the formation or degradation of seed OBs (Lin and Tzen, 2004).

Either within purified OBs from *A. thaliana* seeds or as a purified bacterially expressed chimeric enzyme *in vitro*, AtHSD1 is capable of catalyzing NADP^+^-dependent dehydrogenation of 11β- and 17β- hydroxysteroids, including cortisol, corticosterone, and estradiol, indicating 11β- and 17β- HSD activity. Purified OBs also exhibit NADPH-dependent 17β-ketosteroid reductase activity by which estrone is converted into estradiol. NADP^+^, rather than NAD^+^, is the preferred cofactor for AtHSD1 (d’Andréa et al., 2007). OsHSD1 also displays NADP^+^ and NAD^+^-dependent dehydrogenase activity when using either estradiol or corticosterone as substrates, but shows higher dehydrogenase activity with NAD^+^ than NADP^+^ (Zhang et al., 2016). Steroleosin from *P. massoniana* OBs also exhibits sterol dehydrogenase activity with estradiol and corticosterone as substrates in the presence of NADP^+^ (Pasaribu et al., 2016).

AtHSD1 did not possess 3β-HSD activity as it cannot convert either cholesterol into 4-cholestene-3-one, or dehydroepiandrosterone (DHEA) into 4-androstene-3,17- dione (4AD) under the experimental conditions. Thus, 3β-hydroxysterols are likely not substrates for AtHSD1, suggesting AtHSD1 might belong to the 17β-HSD superfamily (d’Andréa et al., 2007). 3β-HSD activity of HSDs from other plants has also not yet been found.

### HSDs’ Possible Role in Plant Growth and Development

*AtHSD1* expression is tissue specific and is strongly expressed in the above-ground parts of seedlings, especially vascular tissues, and is weakly expressed in root tissues. It has also been observed in the bud and silique pedicels (Li et al., 2007). *AtHSD1* mRNA accumulation dramatically increases during seed and silique maturation, decreasing sharply during late maturation and the early germination process, resulting in full disappearance in fully germinated plantlets and in the vegetative organs and flowers of plants. AtHSD1 protein expression appears to be slightly delayed compared to mRNA accumulation as protein levels remain almost stable during the maturation phase and early germination (Baud et al., 2009). The 5’ region of the *AtHSD1* gene contains two RY motifs that are recognized by LEAFY COTYLEDON2 (LEC2) and FUSCA3 (FUS3) B3 domain proteins (Kroj et al., 2003; Braybrook et al., 2006). These proteins are transcriptional regulators that induce the expression of oleosin, caleosin and certain genes encoding key enzymes involved in fatty acid (FA) biosynthesis and oil accumulation in developing leaves and seeds (Guo et al., 2013; Tang et al., 2018). Therefore, *AtHSD1* tissue specific expression seems to be controlled largely at the transcriptional level, with LEC2 being involved in the transcriptional activation of *AtHSD1*.

Both a loss-of-function *hsd* mutant produced by RNAi and transgenic *Arabidopsis* plants overexpressing *AtHSD1* (AOHSD) have been used to analyze the function of *AtHSD1*. Compared to wild type plants, AOHSD plants show a series of phenotypes including reduced seed dormancy, thicker stems and increased growth, branching, flower production, and seed yield. Similarly, the increased growth phenotype found in AOHSD plants is also observed in transgenic *B. napus* plants overexpressing *AtHSD1*. Accordingly, the *AtHSD1* RNAi mutant (*hsd*) shows a semi-dwarfed phenotype. Together, the phenotypes of AOHSD plants and the *hsd* mutant demonstrate that AtHSD1 is involved in regulating growth and development in plants (Li et al., 2007).

The rice OsHSD1 protein is localized to both the ER and the OB surface and has a similar subcellular localization pattern as AtHSD1. It was found to be expressed in all organs tested including roots, culms, leaves, and panicles, with expression levels highest in leaf sheathes (Zhang et al., 2016). In the rice *oshsd1* mutant, in addition to a reduced plant height phenotype, a thin water film is formed on the leaf surface when the leaves are wetted due to a wax deficiency. Deletion of *OsHSD1* is responsible for the wax-deficient phenotype, providing the first evidence that steroleosin is involved in wax metabolism. The cuticles on the surface of the mutant leaf show a reduced amount of epicuticular wax crystals and thicker cuticle membrane compared to wild type. Further analysis of the wax components showed that long-chain fatty acids (C16 and C18) and VLCFAs (C26, C28, and C30), are significantly increased in the leaves of the mutant. This work provides new insights into HSDs involvement in wax and lipid metabolism.

### Response of AtHSD to BR, ABA, and Abiotic Stress

Besides the phenotypes mentioned above, AOHSD plants also exhibit hypersensitivity to brassinosteroids (BRs), reduced sensitivity to ABA and increased catabolism of ABA (Li et al., 2007). In addition to these, observations also indicate that HSD may be involved in the stress response of plants.

As a powerful plant hormone, BR regulates some growth-specific processes including cell elongation, the promotion of seed germination and plant growth, vascular differentiation, ammonium uptake, photomorphogenesis, and skotomorphogenesis. BR-deficient mutants display extreme dwarf phenotypes (Noguchi et al., 1999). Experimental evidence confirmed that BR promotes seed germination by directly enhancing the growth potential of the emerging embryo (Leubner-Metzger, 2001). Interestingly, *AtHSD1* expression is significantly induced by treatment with BL (brassinolide, the most active BR) in wild type plants but markedly decreased in BR-deficient mutants under similar treatment (Li et al., 2007). Additionally, the increased seed germination, growth and seed yield phenotypes of AOHSD plants are similar to those of plants that overproduce BRs (Choe et al., 2001), overexpress the BR receptor gene *BRI1* (Nam and Li, 2002) or of wild type plants treated with exogenous BL. AOHSD plants also show increased sensitivity to BRs. Accordingly, the *AtHSD1* RNAi mutant (*hsd*) is relatively insensitive to BRs. About 40 genes are significantly induced in AOHSD plants compared to wild type. The function of several these genes are similar to that of BL-induced genes encoding putative cell elongation or expansion-associated proteins, such as pectinesterase and xyloglucan fucosyltransferase (Li et al., 2007). The AOHSD phenotype appears to be due to enhancement of the effect of endogenous BRs or as a result of elevated BR concentrations; therefore, AtHSD is likely responsible for catalyzing a step in the biosynthesis of BRs or is involved in BR signaling (Li et al., 2007).

*AtHSD1* gene expression affects sensitivity to and metabolism of ABA. ABA signaling plays an important role in many biological processes, such as embryo development, seed maturation, dormancy and germination, seedling establishment, vegetative development, root growth, stomatal movement, flowering, pathogen response, senescence, and stress response (Finkelstein, 2013; Wang et al., 2018). After ABA treatment, the levels of all ABA metabolites in AOHSD seeds are much higher than wild type. AOHSD seeds exhibit greatly reduced sensitivity to ABA, while *hsd* mutant seeds are more sensitive to ABA during germination, similar to the BR biosynthetic mutant *det2-1* and the BR-insensitive mutant *bri1-1* (Steber and McCourt, 2001; Li et al., 2007).

Seed germination, plant growth and development are severely affected by various abiotic stresses (Ding et al., 2018; He et al., 2018). Increasing evidence has accumulated demonstrating the interplay of HSDs and stress tolerance. For example, transgenic seeds of both *Arabidopsis* and canola which overexpress HSD (AOHSD and BOHSD) show increased salt tolerance compared to wild type (Li et al., 2007). The expression level of OsHSD1 is induced by NaCl and cold treatment, but is inhibited by drought treatment. Moreover, it is well known that the plant cuticular wax has many functions, including protection against UV radiation, resistance to pathogens and tolerance to environmental stresses (Bruhn et al., 2014; Sun et al., 2015). Studies have also provided some evidence for the relationship between abiotic stress and fatty acid metabolism (Thomas et al., 2012; Zhou et al., 2016; Wang et al., 2017; Sui et al., 2018). The role of OsHSD1 in wax and lipid metabolism and the fact that its expression is induced by NaCl and cold suggest OsHSD1 may be involved in environmental stress responses. However, the exact regulatory mechanism remains to be elucidated. The canola (*B.napus*) homolog of AtHSD1 also shows a putative role in stress response. The protein exhibits higher relative expression in imbibed seeds under polyethylene glycol (PEG) treatment than under treatment with the ABA analog PBI429, and also exhibits higher relative expression in nongerminating, ABA analog-treated seeds than in germinating seeds (Li et al., 2005).

Together, in *A. thaliana*, AtHSD1 has been reported to be important in regulating growth and development, stress tolerance, and produce BR-like effects. However, the genetic mechanisms of the effects and the role of AtHSD1 in response to BR action have not been established, and other HSD homologs have not yet been studied.

## Interactions Between OB-Associated Proteins and Other Proteins

To properly understand the mechanisms of lipid storage regulation, it is essential to define the interactome of OB-associated proteins. However, limited experimental evidence regarding the physical interactions of OB-associated proteins has been obtained, primarily due to technical limitations, especially in plants (Tsai et al., 2015; Kolkhof et al., 2017).

Lipid droplet-associated proteins (LDAPs) are abundant components of OBs in non-seed cell types and are critical for the dynamic regulation of neutral lipid compartmentalization during various developmental and stress-related processes such as heat, cold, and drought conditions. LDAP3 is the most highly and ubiquitously expressed *LDAP* gene in *Arabidopsis*, including in seeds (Gidda et al., 2016). Using the LDAP3 isoform as “bait” to screen a yeast two-hybrid library, Pyc et al. identified a new protein At5g16550, which they named LDIP (LDAP-interacting protein). The protein was confirmed to target specifically to the OB surface with further biochemical and cellular experiments. LDIP T-DNA mutants showed enlarged OBs and had increased total neutral lipid content in both leaves and seeds. These data suggest LDIP is a novel regulator in OB biology (Pyc et al., 2017).

Another case of protein-protein interaction during oleosin degradation in *Arabidopsis* germinated seeds has been mentioned above (Deruyffelaere et al., 2015). Ubiquitination of oleosins occurs at the onset of lipid degradation. Three distinct motifs, including monoubiquitin, K48-linked diubiquitin, and K63-linked diubiquitin, are attached to the major oleosins OLE1 and OLE2. These distinct motifs designate oleosins toward different degradation pathways according to the ubiquitination type. Deruyffelaere et al. further confirmed that PUX10 localized to OBs interacts directly with ubiquitinated oleosins and mediates dislocation of oleosins by the AAA ATPase CDC48A (Deruyffelaere et al., 2018; Kretzschmar et al., 2018).

More work needs to be done to uncover the mechanism of interactions between OB associated proteins and other proteins in OB biology.

## Outlook and Future Perspectives

OB-associated proteins in seeds and the pollen of plants, mainly including oleosin, caleosin, and steroleosin, have been studied extensively in recent years. Although it has been gradually revealed that these proteins possess numerous functions important for cellular physiology, many questions remain.

Regarding OB biogenesis, the mechanisms regulating extraction of OBs to the cytosol and how OBs are recognized and bound by proteins remain incompletely understood. Post-germinative mobilization of neutral lipids stored in seed OBs is preceded by the degradation of OB-anchored proteins. However, the mechanisms underlying the dislocation of these proteins from the OB monolayer are still unknown. It has been known that these proteins are hydrolyzed by endogenous proteases during seed germination and seedling growth. But so far, little information concerning the identity of the endogenous protease has been revealed. In addition to oleosin, caleosin, and steroleosin, several other OB-associated proteins (such as OBAP1 identified in maize scutellum) have been detected recently (López-Ribera et al., 2014; Müller et al., 2017; Kretzschmar et al., 2018; De Chirico et al., 2018). Additionally, distinct populations of OBs have been found to exist in plants, each possessing different proteins, suggesting functional differentiation of OBs in plant seedlings. Further research is needed to study the mechanism of OB dynamics in other plant tissues and to determine what the roles of the newly identified OB-associated proteins in OB biogenesis and turnover may be.

Two OB-associated proteins, caleosin and steroleosin, were confirmed to participate in cellular stress defenses *via* enzymatic activities and hormone signaling, however, the genetic role of these genes in response to stress and hormone action has also not yet been established. For example, AOHSD plants exhibit hypersensitivity to BR, insensitivity to ABA and increased stress tolerance. ABA and BRs have been shown to act antagonistically (Mandava, 1988); thus, it is difficult to reconcile how the BR-like effects of HSD can be associated with enhanced stress tolerance (which is promoted by ABA) (Li et al., 2007). Therefore, the relationship between BR, ABA, and stress tolerance may be more complex and further investigation will be required to define precise mode of action of HSDs in this process.

Moreover, several *CLO* genes or *HSD* genes are closely linked on the chromosomes in *A. thaliana*, so it is difficult to construct multiple mutants using traditional methods. This likely has limited research into caleosin and steroleosin. Now, the CRISPR system (Liang et al., 2018) and traditional crosses may be used to edit several genes simultaneously and construct multiple mutants in which all members of the *CLO* or *HSD* gene families could be knocked out, allowing for further functional analysis.

In short, continuing advances in analytic techniques and genomics will help to find more OB-associated proteins and further reveal the exact role of these proteins in stress response, lipid and BR metabolism, and OB formation. These studies may also provide novel opportunities for increasing stress resistance, enhancing plant yield or increasing the total TAG content in plant tissues for a variety of industrial applications.

## Author Contributions

QS, PW, and CM conceived and wrote the manuscript. XL and TS contributed to the revision of the manuscript. All authors read and approved the submitted version.

## Funding

This study was funded by the Key Technology Research and Development Program of Shandong (2018GSF121037 and 2018GNC113010) and the National Natural Science Foundation of China (31770290 and 31970301).

## Conflict of Interest

The authors declare that the research was conducted in the absence of any commercial or financial relationships that could be construed as a potential conflict of interest.
